# Towards interoperable and reproducible QSAR analyses: Exchange of datasets

**DOI:** 10.1186/1758-2946-2-5

**Published:** 2010-06-30

**Authors:** Ola Spjuth, Egon L Willighagen, Rajarshi Guha, Martin Eklund, Jarl ES Wikberg

**Affiliations:** 1 Department of Pharmaceutical Biosciences, Uppsala University, Uppsala, Sweden; 2 NIH Chemical Genomics Center, 9800 Medical Center Drive, Rockville, MD 20850, USA

## Abstract

**Background:**

QSAR is a widely used method to relate chemical structures to responses or properties based on experimental observations. Much effort has been made to evaluate and validate the statistical modeling in QSAR, but these analyses treat the dataset as fixed. An overlooked but highly important issue is the validation of the setup of the dataset, which comprises addition of chemical structures as well as selection of descriptors and software implementations prior to calculations. This process is hampered by the lack of standards and exchange formats in the field, making it virtually impossible to reproduce and validate analyses and drastically constrain collaborations and re-use of data.

**Results:**

We present a step towards standardizing QSAR analyses by defining interoperable and reproducible QSAR datasets, consisting of an open XML format (QSAR-ML) which builds on an open and extensible descriptor ontology. The ontology provides an extensible way of uniquely defining descriptors for use in QSAR experiments, and the exchange format supports multiple versioned implementations of these descriptors. Hence, a dataset described by QSAR-ML makes its setup completely reproducible. We also provide a reference implementation as a set of plugins for Bioclipse which simplifies setup of QSAR datasets, and allows for exporting in QSAR-ML as well as old-fashioned CSV formats. The implementation facilitates addition of new descriptor implementations from locally installed software and remote Web services; the latter is demonstrated with REST and XMPP Web services.

**Conclusions:**

Standardized QSAR datasets open up new ways to store, query, and exchange data for subsequent analyses. QSAR-ML supports completely reproducible creation of datasets, solving the problems of defining which software components were used and their versions, and the descriptor ontology eliminates confusions regarding descriptors by defining them crisply. This makes is easy to join, extend, combine datasets and hence work collectively, but also allows for analyzing the effect descriptors have on the statistical model's performance. The presented Bioclipse plugins equip scientists with graphical tools that make QSAR-ML easily accessible for the community.

## Background

Quantitative Structure-Activity Relationship (QSAR) modeling is a ligand-based approach to quantitatively correlate chemical structure with a response, such as biological activity or chemical reactivity. The process is widely adopted and has for example been used to model carcinogenecity [1,2], toxicity [3,4], and solubility [5,6]. Further, the literature is replete with QSAR studies covering problems in lead optimization [7], fragrance design, and detection of doping in sports [8]. In QSAR, chemical structures are expressed as descriptors, which are numerical representations such as calculated properties or enumerated fragments. Descriptors and response values are concatenated into a dataset, and statistical methods are commonly used to build predictive models of these.

There exist many examples of investigations regarding the resulting statistical models with respect to validity and applicability in QSAR and similar fields [9,10]. However, most of these investigations consider the dataset as fixed, and the choice of descriptors and implementations is left outside the analysis.

Part of the problem is the lack of a controlled vocabulary regarding descriptors; there is no easy way of defining what descriptors were used, which the underlying algorithms were, and how these were implemented. It is common to use several different software packages with results manually glued together in spreadsheets, sometimes with custom in-house calculated descriptors. The lack of a unifying standard and an exchange format means that QSAR datasets are published in articles without clear rules, usually as data matrices of precalculated descriptors, with chemical structures in a separate file.

The field of bioinformatics has acknowledged the standardization problem to a much larger extent than cheminformatics. Numerous standards, ontologies, and exchange formats have been proposed and agreed upon in various domains. The Minimum Information standards are examples that specify the minimum amount of meta data and data required to meet a specific aim. The MGED consortium pioneered this in bioinformatics with Minimum Information About Microarray Experiements (MIAME) [11], and it has now become a requirement that data from microarray experiments must be deposited in MIAME-compliant public repositories in the MAGE-ML exchange format [12], in order to be published in most journals. Standardization initiatives in cheminformatics are not as common, even though the problem of incompatible file formats and standards has been frequently discussed [13]. Grammatica [14] has addressed the issue of QSAR model validation and notes that descriptor versioning as well as precisely defined algorithmic specifications are vital for developing QSAR models that can be considered reliable, robust, and reproducible (in addition to the usual issues of statistical rigor).

Initiatives that work towards standardizing cheminformatis in general include the Blue Obelisk, an internet group which promotes open data, open source, and open standards in cheminformatics [15], which has proposed dictionaries for algorithms and implementations suitable for QSAR. Distributed Structure-Searchable Toxicity (DSSTox) Database Network has proposed standardized structure-data files (SDF) as a file format for exchanging raw data in toxicological SAR analyses [16]. This approach does however not include any information regarding descriptors, and SDF is a legacy text format which has many variants. OECD has established rules and formats for how to report QSAR models and QSAR predictions [17], but its intended use is communication, not complete technical coverage. It also lacks an ontology, which makes interpretation and reasoning around results much more complicated and subjective. Public repositories of QSAR datasets are limited to a few internet resources (e.g. [18] and [19]) where they are usually not deposited but reproduced from articles by others than the original authors, and due to the lack of an established exchange format and missing raw data, structures are sometimes redrawn, data manually copied from articles, and educated guesses are made in some cases. QSAR DataBank [20] is a proposal for the electronic organization and archiving of QSAR model information. It is an interesting initiative that builds on other standards, but also lacks an ontology for descriptors. The OpenTox project is another project developing a framework to share QSAR datasets using REST services [21].

In general it is not uncommon that information about what software package that was used for descriptor calculation (and its version) is unavailable, and that custom descriptors have been added manually or results preprocessed. To further complicate matters, many QSAR software packages are proprietary, closed source, and it is a non-trivial task (sometimes impossible) to get insights into how algorithms are implemented. Due to these impracticalities, journals are limited to establishing simple rules for QSAR publications such as to state that structures should be publicly available [22].

A well-defined standard with a corresponding exchange format will have problems getting accepted in the scientific community if user-friendly tools supporting them are not available. This paper introduces a file format for exchanging QSAR datasets, together with tools implemented in the graphical workbench Bioclipse [23,24] to facilitate working with QSAR according to the standard.

## Results

### QSAR-ML - an exchange format for QSAR

**Figure 1:**
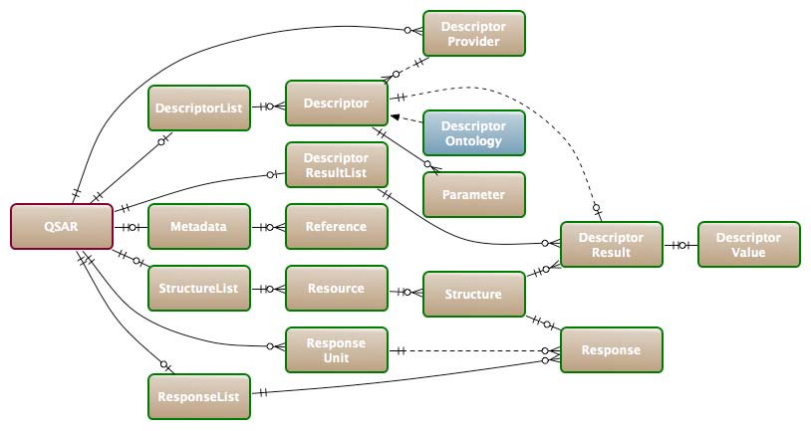
**QSAR-ML core structures**. An simplified diagram of the structure of QSAR-ML using Crow's Foot notation, with references using dotted lines.

We designed an XML-based exchange format (named QSAR-ML) with the aim to completely cover all aspects of dataset setup, including chemical structures, descriptors, software implementations, and response values. A simplified structure of QSAR-ML can be seen in Figure 1.

*Structures *define the chemical structures in the QSAR dataset and contains InChI [25] to ensure integrity; if a structure changes, then the QSAR-ML file can report this. *Structures *are referenced parts of a *Resource*, which is a file referenced by path or URL and also contains a checksum that can be used to verify the integrity of the files. *Resources *are in turn contained in a *StructureList*.

*Descriptors *are uniquely defined by referencing the Blue Obelisk Descriptor Ontology (BODO) [26] and are contained in a *DescriptorList *. A *Descriptor *can also have a set of *Parameters*, which for example can be settings for the descriptor. A *DescriptorProvider *denotes a versioned software implementation, which provide implementations of descriptor algorithms.

*Responses *are the measured QSAR endpoints (response variable). They reference a *Structure *and a *ResponseUnit *(for example IC_50 _or LD_50_), and are contained in a *ResponseList*.

*DescriptorResults *are the results of a descriptor calculation on a structure, and links a *DescriptorValue *to a *Descriptor-Structure *pair. *DescriptorResults *are contained in a *DescriptorResultList*.

*Metadata *includes information about authors, license, description, and also contains optional *References*. The latest version of the QSAR-ML schema and documentation is available from the QSAR-ML website [27].

### Reference Implementation

**Figure 2:**
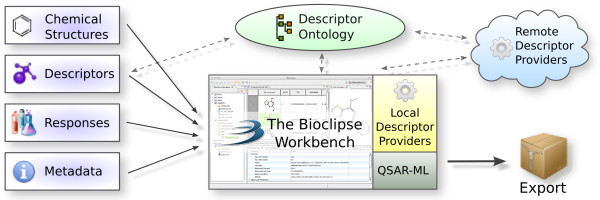
**Overview of the Bioclipse QSAR-ML implementation**. The reference implementation of QSAR-ML is constructed as a set of plugins for Bioclipse and allows for graphical setup of datasets. Chemical structures can be imported via drag and drop or a graphical wizard. Descriptors can be selected from the descriptor ontology. Local and remote descriptor providers contribute descriptor implementations which could run on the local computer or accessed via Web services. It is also possible to add biological responses and metadata, and export the complete dataset in QSAR-ML as well as in a comma-separated file.

While QSAR-ML is technology neutral, a reference implementation of tools to set up QSAR datasets complying with QSAR-ML was constructed as a set of plugins for Bioclipse [23]. The implementation allows for straightforward creation, loading, saving, editing, and export of QSAR-ML files (see Figure 2).

**Figure 3:**
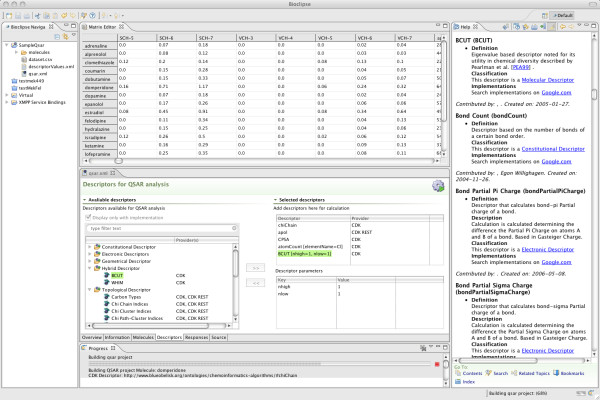
**Selecting descriptors from the Blue Obelisk Descriptor Ontology in Bioclipse**. Screenshot from Bioclipse showing selection of descriptors (lower middle), the generated dataset in a spreadsheet (top middle), the Help View (right) showing interactive help for descriptors, and the Progress View indicating the progress of the descriptor calculations (lower bottom).

Using graphical wizards and drag and drop, users can easily set up new QSAR analyses, add molecules, select descriptors and implementations with optional parameters, import or add response values, and the calculations can be carried out in the background (see Figure 3). It is very easy to export QSAR-ML for import in other QSAR-ML compliant software.

The Bioclipse-QSAR feature supports multiple descriptor providers; the only requirement is that the software must be able to accept one or many chemical structures, and deliver descriptors in a deterministic fashion that can be accessed either programmatically or via a batch job (e.g. shell script). Bioclipse-QSAR also support calling descriptor calculations deployed as W3C Web services [28], REST [29], and XMPP cloud services [30]. To add new descriptors to Bioclipse, the descriptor should preferably be registered in the Blue Obelisk Descriptor Ontology, but it could also be added to Bioclipse via a separate file.

Bioclipse-QSAR comes with the Chemistry Development Kit (CDK) [31] and JOELib [32] integrated as local descriptor providers, supplying descriptor implementations with optional parameters that are run in the same computer and hence do not require network connection. Remote Web services of the CDK descriptors are available as REST [33] and XMPP services (see Methods section). It is also possible to use the QSAR feature in the Bioclipse Scripting Language [34] for setting up datasets.

The Bioclipse-QSAR feature is available via the software update menu option in Bioclipse, from the main Bioclipse Update Site. Bioclipse and the Bioclipse-QSAR feature are released under Eclipse Public License [35] plus an exception to allow GPL-licensed Bioclipse plugins (see [23]). EPL is a flexible open source license that can be extended by both open source as well as commercially licensed plugins.

### Sample datasets

For demonstration purposes, the chemical structures for a subset of the Sutherland datasets [36] were subjected to descriptor calculations for selected CDK descriptors and are available in QSAR-ML and archived Bioclipse projects at the QSAR-ML website [27].

## Discussion

The QSAR-ML exchange format together with the Blue Obelisk Descriptor Ontology has many implications. To the best of our knowledge it is the first initiative which encompasses completely reproducible definition of QSAR datasets, including descriptor definitions and implementations. QSAR-ML is equipped with built-in properties to ensure integrity and consistency of included resources. For example are molecular resources appended with generated InChI, which can be used to verify the integrity of the chemical structures such as accidental changes or errors when transmitting data over networks. That descriptors in QSAR-ML are defined in Blue Obelisk Descriptor Ontology means that they have a formalized and clear meaning, and are uniquely referenced. Defining descriptor implementations by software name, version and identifier, and connecting this information with an entry in the descriptor ontology, uniquely defines, and makes it possible to accurately reproduce descriptor calculations. Open standards, a defined terminology, and reproducible results allows people to have trust in publicly available datasets and reconstruction of such datasets, and hence improves the reliability of the subsequent statistical analysis. Much research has been done on various aspects of QSAR modeling, such as validation, robustness, and domain applicability of models [14]. This is not covered here as it is a research topic of its own, but we stress that the handling of the original chemical structures as well as the choice and implementation of descriptors are of great importance [37]. This is a neglected topic, and QSAR-ML sets new standards for the field. A reproducible dataset setup enables validation not only of the resulting datasets, but allows for inclusion of e.g. chemical variability and descriptor selections with respect to model robustness and performance inside a cross validation loop [10]. There is a large amount of descriptors available, and people continuously improve existing descriptors and develop new ones. An exchange format capable of harmonizing this requires an extensible architecture in order to be successful, and also intuitive tools that make this easily available for scientists. QSAR-ML, implemented as an XML Schema, and BODO, implemented in the Web Ontology Language [38], fulfills this demand of extensibility. We would like to point out that this is a proposal for an open standard and that we welcome suggestions to improve the specification further.

The Bioclipse-QSAR feature turns Bioclipse into a workbench which greatly simplifies the setup of QSAR datasets, with full support for the QSAR-ML exchange format. Bioclipse also supports many other features which are common in QSAR projects, such as conversion, editing, and visualization of chemical structures. Rich clients are software applications that take full advantage of today's modern desktop computers, but also leverages on the new e-Science tools such as online (Web) services. The Bioclipse-QSAR is a formidable example of this; Descriptors can be calculated on the local computer while, if connected to a network, remote services can provide additional descriptors or offer high performance computers for speeding up demanding calculations.

There would be great rewards if QSAR-ML is widely adopted by the scientific community. For example, users could download entire QSAR datasets and reuse it together with in-house data, extend existing models, join different models, search for overlap between datasets, collaborate, reproduce, and validate results. Further, QSAR-ML enables the establishment of public repositories of QSAR datasets. We envision that deposition of QSAR models in such repositories will become a standard operation procedure prior to future publication of QSAR models and results, similar to microarray experiments in bioinformatics, and that QSAR-ML is a strong candidate for such a format.

## Conclusions

We describe a new exchange format for QSAR datasets, named QSAR-ML, which relies on the Blue Obelisk Descriptor Ontology for uniquely defining descriptors, and supports any implementation of these. QSAR-ML comprises all data and metadata required to reproduce the setup of QSAR datasets, enabling validation of chemical structures and descriptor calculations. Sharing QSAR datasets in an open, standardized format has profound implications for collaboration and information validity and reuse. We also describe a QSAR plugin for Bioclipse with full support for QSAR-ML, which greatly simplifies setting up QSAR datasets using graphical user interfaces. The implementation integrates with other cheminformatics component that are valuable in dataset preparation, such as database searching as well as editing and visualization of chemical structures.

Future plans include addition of subsequent statistical analysis into the QSAR-ML format and hence not only support dataset setup but also model fitting and prediction. We also aim at setting up a public repository with means for publishing QSAR-ML datasets, which is a first step towards public repositories for sharing QSAR on a global level, and could provide the basis for supplemental data in future QSAR publications.

## Methods

### XML Schema

XML is an extensible markup language that is widely used in bioinformatics as an easy to use and standardized way to store self-describing data [39]. W3C XML Schema [40] is used in this work to define rules in QSAR-ML, such as required elements and data types. It can also be used to validate an XML document to ensure that it conforms to the rules. The latest version of the QSAR-ML schema together with documentation is available on the QSAR-ML website [27].

### Bioclipse

Bioclipse [23] is a graphical workbench for life science which is equipped with features required for many common cheminformatics tasks, such as loading and converting between file formats, editing of chemical structures, interactive visualisation in 2D/3D, and editing of compound collections. Bioclipse is implemented as a Rich Client based on Eclipse [41], and is equipped with advanced plugin architecture which makes it easy to add new descriptor providers (for example third party software or custom implementations), and allows users to cherry-pick descriptors and implementations for the current analysis (see Figure 3).

### CDK descriptors

The Chemistry Development Kit (CDK) [31] aims to provide a comprehensive collection of descriptors [42]. In contrast to many other packages, the CDK provides descriptors for molecules, bonds and atoms. While most QSAR analyses make use of molecular descriptors, the presence of the other descriptor types allows for novel approaches to QSAR modeling. Given that many thousands of descriptors have been described in the literature [43], CDK is focused on descriptors that have been used in numerous studies. Many of these descriptors derive from the ADAPT package [44]. Broadly, the descriptors can be categorized into four main groups: constitutional (which consider various atom and bond counts), topological (which consider 2 D connectivity), geometric (which consider the 3 D spatial arrangement of a molecule) and electronic (which consider electronic properties of the molecule). There are a total of 44 descriptor classes. It should be noted that each descriptor may actually generate multiple values. Thus the total number of descriptor values that can be calculated is much higher (in the order of 280 descriptors).

### JOELib descriptors

JOELib is an open source Java cheminformatics library [32]. A Bioclipse plugin for JOELib was constructed and provides ten QSAR descriptors, some of which overlap with the CDK descriptors. However, JOELib also provides a few unique descriptors, including a LogP descriptor implementing an atomic contribution algorithm [45] and two SMARTS-based fragment count descriptors counting the number of acidic and basic groups.

### Remote REST and XMPP services

REST services for CDK descriptors [33] are available from http://ws1.bmc.uu.se:8182/cdk/descriptors, which conforms to REST principles [29]. The return values from the REST services are in a custom XML format which is very minimal and thus extraction of descriptor values is trivial. The REST based services result in much simpler programmatic access and reduce the number of dependencies in client code than for example SOAP services [28]. XMPP cloud services with IO-DATA [30] is a novel technology that allows for discoverable, asynchronous Web services. XMPP services for calculating several CDK descriptors are available from the XMPP server http://ws1.bmc.uu.se.

## Competing interests

The authors declare that they have no competing interests.

## Authors' contributions

OS and ME designed the QSAR-ML. EW and RG designed and implemented the BODO. OS implemented the Bioclipse-QSAR plugins. EW implemented the JOELib plugin. JW supervised the project. All authors read and approved the final manuscript.

## Acknowledgements

The authors would like to thank all the people who have contributed to the Blue Obelisk Descriptor Ontology and the Bioclipse project, as well as Anders Lövgren at the computing department at Uppsala Biomedical Center (BMC) for hosting the CDK REST and XMPP services.

This work was supported by the Swedish VR (04X-05957) and Uppsala University (KoF 07).
